# Quantitative analysis of the epithelial lining architecture in radicular cysts and odontogenic keratocysts

**DOI:** 10.1186/1746-160X-2-4

**Published:** 2006-02-17

**Authors:** Gabriel Landini

**Affiliations:** 1 Oral Pathology Unit. School of Dentistry, The University of Birmingham, St. Chad's Queensway, Birmingham B4 6NN, UK

## Abstract

**Background:**

This paper describes a quantitative analysis of the cyst lining architecture in radicular cysts (of inflammatory aetiology) and odontogenic keratocysts (thought to be developmental or neoplastic) including its 2 counterparts: solitary and associated with the Basal Cell Naevus Syndrome (BCNS).

**Methods:**

Epithelial linings from 150 images (from 9 radicular cysts, 13 solitary keratocysts and 8 BCNS keratocysts) were segmented into theoretical cells using a semi-automated partition based on the intensity of the haematoxylin stain which defined exclusive areas relative to each detected nucleus. Various morphometrical parameters were extracted from these "cells" and epithelial layer membership was computed using a systematic clustering routine.

**Results:**

Statistically significant differences were observed across the 3 cyst types both at the morphological and architectural levels of the lining. Case-wise discrimination between radicular cysts and keratocyst was highly accurate (with an error of just 3.3%). However, the odontogenic keratocyst subtypes could not be reliably separated into the original classes, achieving discrimination rates slightly above random allocations (60%).

**Conclusion:**

The methodology presented is able to provide new measures of epithelial architecture and may help to characterise and compare tissue spatial organisation as well as provide useful procedures for automating certain aspects of histopathological diagnosis.

## Introduction

Odontogenic cysts of the jaws include various pathological entities. By definition, these are cysts (i.e. pathological cavities with fluid or semi-fluid contents but excluding pus) with an epithelial lining that derives from the tooth-forming organ epithelia: the so-called *glands of Serres *(rests of the *dental lamina*), the rests of Malassez (rests of the *root sheath of Hertwig*) and the *reduced enamel epithelium *(remnants of the *enamel organ *after dental crown formation) – although for odontogenic keratocysts it has also been proposed that the lining may derive from mucosal basal cells [12]. The aetiology of these lesions has been traditionally classed into two different groups: developmental (dentigerous, keratocysts, gingival cysts, etc.) and inflammatory (radicular, residual, paradental cysts). In terms of their incidence, radicular cysts are the commonest (mostly associated to teeth with pulp necrosis due to advanced dental caries), followed by dentigerous and odontogenic keratocysts (OKs) [12].

Some types of odontogenic cysts have characteristic epithelial linings and differ in their behaviour. While most epithelial cysts are thought to grow passively driven by hydrostatic pressure inside the lumen created by the hypertonic cyst fluid content (mostly epithelial desquamation debris) which is maintained by the semi-permeable epithelial lining, other cysts show active cellular proliferation, therefore, for diagnostic purposes, it is important to characterise quantitatively the differences across the different entities.

In relation to OKs, there are two significant diagnostic issues. Firstly, they commonly show active epithelial grow which has prompted the belief that they should perhaps be regarded as neoplasms rather than cysts. This seems to be supported by the observation that the epithelial cells in the lining of these lesions possess genetic abnormalities in specific tumour suppressor genes [1]. Secondly, they are known to occur in two fashions: solitary (or sporadic) and as part of the Basal Cell Naevus (or Gorlin-Goltz's) Syndrome (BCNS). This syndrome is an autosomal dominant condition with complete penetrance and variable expressivity, characterised by the presence of multiple nevoid basal cell carcinomas of the skin, multiple (synchronous or metachronous) odontogenic keratocysts of the jaws, skeletal abnormalities, ectopic calcifications and plantar or palmar pits. The diagnosis of an OK is therefore an important clue that should flag the need to further examination of other BCNS signs. This has prompted questions about whether it is possible to differentiate between the two subtypes of keratocyst at the histomorphological level. Expert opinions on this subject seem contradictory [2]. While some authors have reported significant differences between solitary and BCNS OKs, the possibility of discrimination at a statistical level for diagnostic (classification) purposes has not been addressed to provide a definitive answer.

Therefore, in this paper, the analysis was directed to elucidate this problem by studying 1) the architectural differences between two main types of odontogenic cysts: radicular cysts and keratocysts, and 2) between the solitary and BCN syndrome keratocyst subtypes.

This was investigated by means of image processing techniques applied to digitised histological images of cysts using a systematic spatial discretisation of the cellular elements in the epithelial lining. To this end, a method for theoretical cell segmentation in the epithelial compartment was applied, followed by an algorithmic grouping of the resulting cells into "layers". Finally a morphometric analysis of the segmented cells (indexed by the layer they belong to) was applied to allow statistical comparisons and discrimination rates across the different pathological classes.

## Materials and methods

The material of this study consisted of 5 μm thick sections stained with haematoxylin and eosin (H&E) from formalin fixed and paraffin embedded specimen from the histological archives of the Oral Diagnostic Service at the University of Birmingham.

The samples included 9 cases of radicular cysts (Male:Female ratio 5:4, mean age 41 years ± 18), 13 solitary keratocysts (without inflammatory infiltration) (Male:Female ratio 5:1, mean age 35 years ± 18) and 8 different keratocysts from 5 patients with the BCNS (also without inflammatory infiltration) (Male:Female ratio 2:3, mean age 20 years ± 3). For each case, 5 non-overlapping images with intact epithelial lining and with no apparent oblique direction of sectioning were captured (total: 150 images). Images were digitized using a Olympus BX50 microscope (Olympus Optical Co. Tokyo, Japan) with ×40 objective UPLanFl (resolution: 0.45 μm) at a size of 768 × 572 pixels (resolution: 0.31 μm). A colour camera JVC KY-55B 3-CCD (JVC, Tokyo, Japan) was attached to a 24 bit RGB frame grabber (Imaging Technologies IT4PCI, Bedford, MA, U.S.A.) and controlled by Optimas version 6.51 (Media Cybernetics, Silver Spring, MD, U.S.A.) software running on a standard personal computer. The images were the average of 32 consecutive shots (to reduce camera noise) and they were corrected by computing ratio of the image with a 32-frame averaged background illumination field (to compensate uneven background illumination and the filament colour temperature) minus a 32-frame averaged non-illuminated frame (to compensate for CCD electronic bias). Subsequent imaging procedures were performed using ImageJ version 1.34 (a multiplatform, free and open-source imaging program written by W. Rasband at the NIH, USA) [9]. The analytical procedures were either written in ImageJ's internal macro scripting language or as "plugin" modules for ImageJ written in the Java computer language (Sun Microsystems Inc., Santa Clara, USA).

### Cell profile segmentation

Under light microscopy of H&E stained sections it is not possible to consistently define the limits between adjacent epithelial cells. Instead, theoretical cell profile extents were approximated using a space partition procedure. This has been described in detail elsewhere [6,7]. Briefly, the segmentation is achieved in two steps: 1) nuclear localization based on the optical density of the histological stain, followed by 2) a spatial partition of the epithelial compartment into exclusive areas of influence of each nucleus profile. The nuclear localization (step 1) was determined by isolating the haematoxylin stained areas with the colour deconvolution algorithm developed by Ruifrok & Johnston [11]. The "deconvolved" image retains only the spatial localization of nucleic acids and thus the nuclear locations can be readily extracted. Since epithelial cells are also rich in RNA, their cytoplasms also retain some (albeit less intense) haematoxylin staining and therefore the whole epithelial compartment can also be isolated by optical intensity thresholding (therefore segmented from the underlying connective tissue and the empty lumen).

The spatial partition (step 2) divides the epithelial compartment into exclusive "areas of influence" or "catchment basins" relative to each nucleus (so each area is associated with only one nucleus) by means of an image processing computation called the watershed transform [13]. These areas represent, in theory, the individual epithelial cell profile extents and are based on the nuclear locations and are referred to as 'cells' in the rest of this paper. Those pixels that cannot be assigned to a unique catchment basin are called "watershed lines" and represent the boundaries between cells.

**Figure 1:**
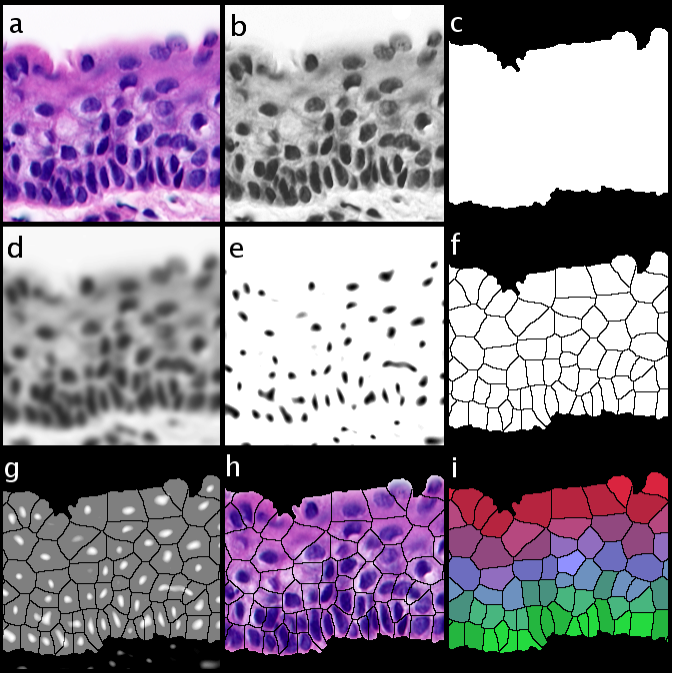
The sequence of procedures to segment the epithelial tissue space into theoretical cell profiles. **a**) original, **b**) optical density of the Haematoxylin stain after colour deconvolution. **c**) the epithelial compartment, **d**) a smoothed version b) after 4 passes of an averaging filter of kernel size 5 pixels, **e**) morphological basins, **f**) catchment basins (theoretical cell profiles) after applying the watershed transform, **g**) average of the negative of e) and f) to show that each "morphological basin" determines a "catchment basin" area. **h**) logical AND operation of f) and a) to visualise the result of the segmentation. Image **i**) shows the layers of the tissue labelled as RGB triplets intensity according to their distance from 3 different references (basal layer (red), superficial layer (green) and both layers (blue)).

Figure 1 presents the most relevant steps in the sequence of procedures leading to the proposed image segmentation. Figure 1a is the original image while 1b shows the optical density contribution of the Haematoxylin stain alone after colour deconvolution. In Figure 1c is shown the epithelial compartment of 1b obtained by histogram equalisation, binary thresholding, hole filling and image cleaning (deletion of all thresholded objects except the largest one). Frame 1d is a smoothed version of 1b after 4 passes of an averaging filter of kernel size 5 pixels to retain only large scale features of the nuclei. Image 1e shows the nuclear localisation by the extraction of the so-called "morphological basins" (or domes, depending whether they are bright or dark). These basins are connected regions in the image of a chosen "depth" in the greyscale function, measured from their deepest (darkest) part upwards, (or *vice versa *for domes). This procedure brings the dark image areas with different optical densities to nearly-equal levels (note that not all the nuclei in 1b are not equally dark). In Figure 1f are shown the catchment basins (theoretical cell profiles) after applying the watershed transform to image 1e (using the watershed plugin written by D. Sage available at ). Image 1f (the average of the negative of 1e and 1f) shows that each "morphological basin" determines a "catchment basin" area. Image 1h is the logical AND operation of 1f and 1a to visualise the result of the segmentation. Image 1i displays the different layers of the tissue labelled as RGB triplets intensity according to their distance from 3 different references (basal layer (red), superficial layer (green) and both layers (blue)).

**Figure 2:**
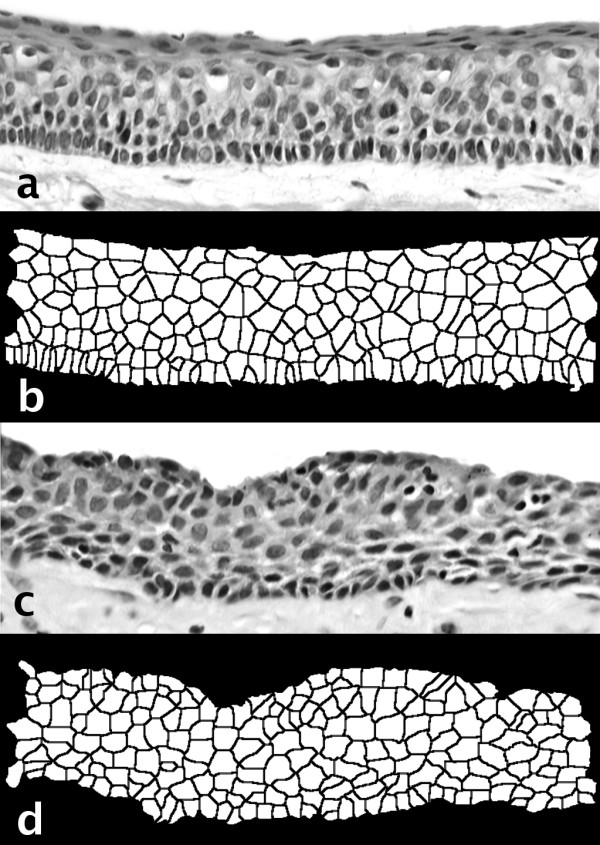
Two examples of the theoretical cell segmentation process. From top to bottom: **a**) a solitary odontogenic keratocyst lining with **b**) its cell segmentation image, **c**) a radicular cyst lining and **d**) its cell segmentation image. Note the palisading in the keratocyst and the variable epithelial thickness of the radicular cyst.

Figure 2 shows epithelial lining profiles and the corresponding segmented sets.

### Layer level estimation

After the partitioning, the layer level of each cell was determined with a distance transform method suitable for non-regular lattices [6,7] where the distance (in layers) can be estimated from any arbitrary reference point. Here the underlying connective tissue was used as reference, so the first layer corresponded to the basal cells, the second layer to the parabasal layer and so on for the remaining epithelium (i.e. "counting up" from the basal layer to the superficial layer).

### Morphometrical analysis

A total of 27 morphological parameters (11 native geometrical measures and 16 derived from various combinations) were extracted from the cells (listed in Table 1). Among these parameters, the longest axis of the cell (called Feret diameter) and its angle of orientation were extracted. This angle is relative to the measuring coordinate system (i.e. the angle is useful when considered in relation to a fixed reference). However, because the coordinate reference in an image with respect to the tissue is somewhat arbitrary, an internal reference relative to the tissue was computed following the direction of the cell layer in which the cell is located. Otherwise, undulating rete ridges and positioning of the specimen in the image would make the orientation measurements meaningless (they would depend on specimen orientation). The local layer orientation reference was estimated for each cell based on the direction of their nearest neighbouring cells within the layer (the direction of the group of cells that include the current cell in question, its nearest neighbours and the next-to-nearest neighbours). The angle of the maximum Feret diameter of the cell in question was then offset to the local orientation of the layer. Full details of this technique with examples have been published elsewhere [6].

**Table 1:** Morphometrical parameters used in the analysis of the theoretical cells.

**Parameter**	**Units**	**Explanation**
Perim	pixels	Perimeter calculated from the centres of the boundary pixels
Area	pixels^2^	The area inside the polygon defined by the perimeter
MinR	pixels	Radius of the inscribed circle centred at the centre of mass
MaxR	pixels	Radius of the enclosing circle centred at the centre of mass
Feret	pixels	Largest axis length
Breadth	pixels	The largest axis perpendicular to the Feret diameter
CHull	pixels	Convex Hull or convex polygon calculated from pixel centres
CArea	pixels^2^	Area of the Convex Hull polygon
MBCRadius	pixels	Radius of the Minimal Bounding Circle
AspRatio	none	Aspect Ratio = Feret/Breadth
Circ	none	Circularity = 4*π*Area/Perimeter^2^, also called form factor
Roundness	none	Roundness = 4*Area/(π*Feret^2^)
AreaEquivD	pixels	Area of circle with equivalent diameter = sqrt((4/π)*Area)
PerimEquivD	none	Perimeter of circle with equivalent diameter = Area/π
EquivEllipseAr	pixels^2^	Equivalent Ellipse Area = (π*Feret*Breadth)/4
Compactness	none	Compactness: sqrt((4/π)*Area)/Feret
Solidity	none	Solidity = Area/Convex_Area
Concavity	pixels^2^	Concavity = Convex_Area - Area
Convexity	none	Convexity = Convex_Hull/Perimeter
Shape	none	Shape = Perimeter^2^/Area
RFactor	none	RFactor = Convex_Hull/(Feret*π)
ModRatio	none	Modification Ratio = (2*MinR)/Feret
Sphericity	none	Sphericity = MinR/MaxR
ArBBox	pixels^2^	ArBBox = Feret*Breadth, area of the box along Feret diameter
Rectang	none	Rectangularity = Area/ArBBox

Here, the "pixel" units relate to length (the distance between the centre of one pixel and its neighbour), while pixels^2 ^relate to area (the number of pixels). "None" are dimensionless values.

ImageJ plugins to perform some of the steps described (morphometrical analysis, morphological dome extraction and colour deconvolution) are currently available from: .

Statistical analysis of the data was done using SPSS version 10 (SPSS Inc., Chicago, USA). Because there is a possibility of correlations between parameters (specially those which are derived from combinations of the native ones), stepwise discriminant analyses were performed. This kind of analysis discards parameters that do not improve the classification rates (likely to be correlated with other parameters). When comparing groups, statistical differences with a probability value less than 0.05 were considered significant.

## Results

Out of the 150 images, a total of 12,853 solitary keratocyst cells, 7,238 BCNS keratocysts cells and 7,715 radicular cyst cells were segmented (total 27,806).

### Cell-wise comparisons

A Multivariate General Linear Model analysis revealed that the mean values of the morphological parameters were statistically different when considering cyst type as a factor (p < 0.001). *Post-hoc *pairwise comparisons with Tukey's tests (revealing any homogeneous subsets) disclosed that the mean of great majority of parameters were statistically different (shown in Table 2).

**Table 2:** Mean morphometrical parameter values in the three cyst types and their pairwise comparisons.

**Parameter**	**Solitary OK (± SD)**	**BCNS OK (± SD)**	**Radicular (± SD)**
Perim	100.5313 (± 26.4673)	103.2415 (± 27.1678)	108.1265 (± 34.1349)
Area	553.1672 (± 295.9194)	583.5653 (± 302.4889)	641.1525 (± 424.4672)
MinR	8.3662 (± 2.8550)	***8.6381 (± 2.8448)***	***8.6490 (± 3.2781)***
MaxR	19.5292 (± 5.3661)	20.0227 (± 5.5363)	21.1478 (± 6.8432)
Feret	36.4066 (± 9.9051)	37.2535 (± 10.1607)	39.2035 (± 12.4571)
Breadth	25.1631 (± 7.3007)	25.9255 (± 7.3639)	26.6557 (± 8.9150)
CHull	95.1866 (± 24.3340)	97.6547 (± 24.9523)	102.1490 (± 31.1970)
CArea	612.2418 (± 329.0827)	646.1540 (± 336.4255)	724.8820 (± 486.0023)
MBCRadius	18.3400 (± 4.9405)	18.7778 (± 5.0672)	19.7536 (± 6.2371)
AspRatio	1.5057 (± 0.4076)	1.4883 (± 0.3963)	1.5276 (± 0.4122)
Circ	***0.6520 (± 0.096)***	***0.6540 (± 0.0939)***	0.6411 (± 0.0988)
Roundness	0.5143 (± 0.1187)	0.5196 (± 0.1171)	0.5031 (± 0.1166)
AreaEquivD	25.6956 (± 6.6373)	26.4050 (± 6.7678)	27.3257 (± 8.3459)
PerimEquivD	32.0001 (± 8.4248)	32.8628 (± 8.6478)	34.4177 (± 10.8655)
EquivEllipseAr	756.3853 (± 401.9763)	797.0583 (± 413.0425)	884.5156 (± 583.8462)
Compactness	0.7120 (± 0.0859)	0.7158 (± 0.0846)	0.7042 (± 0.0853)
Solidity	0.9034 (± 0.0520)	0.9037 (± 0.0516)	0.8877 (± 0.0600)
Concavity	59.0746 (± 53.9079)	62.5887 (± 54.9009)	83.7295 (± 88.4315)
Convexity	0.9485 (± 0.0172)	***0.9476 (± 0.0183)***	***0.9472 (± 0.0231)***
Shape	***19.7841 (± 3.6785)***	***19.7227 (± 3.7068)***	20.1933 (± 4.0637)
RFactor	0.8365 (± 0.0546)	0.8391 (± 0.0545)	0.8337 (± 0.0553)
ModRatio	0.4699 (± 0.1315)	0.4748 (± 0.1299)	0.4523 (± 0.0131)
Sphericity	0.4393 (± 0.1264)	0.4430 (± 0.1247)	0.4210 (± 0.1256)
ArBBox	963.0597 (± 511.8121)	1014.8461 (± 525.9020)	1126.2003 (± 743.3760)
Rectang	***0.5764 (± 0.0652)***	***0.5770 (± 0.0656)***	0.5723 (± 0.0675)

OK: odontogenic keratocyst, BCNS: basal cell naevus syndrome, SD: standard deviation from the mean.

N = 27,806. Mean values across columns are statistically different, except for those in the ***bold ***(General Linear Model with *post-hoc *Tukey, p < 0.05)

A hierarchical stepwise discriminant analysis using all the cell morphological parameters (without taking into account the cell layer position in the epithelium) revealed that 42% of cells could be classified correctly into their original classes (solitary OK, syndrome OK or Radicular cyst). This rate is higher than by random allocation (33%). However the classification rate between the two subtypes of OKs was only 53% and between the pooled OKs and radicular cysts was 66% (random allocation = 50%).

Initially, this seems to indicate that there is little or no information provided for discrimination purposes by the morphological analysis. However, it could be possible that positional (architectural) information associated to the morphological revealed further differences. To investigate this possibility, the analysis was repeated, but considering each layer of the epithelium as a group to allow layer-wise comparisons across the 3 classes (described in the following section).

### Layer-wise comparisons

The mean number of layers case-wise was 8.5 ± 1.7, 7.8 ± 3.1 and 11.4 ± 5.3 for the solitary OKs, syndrome OKs and radicular cysts respectively; ANOVA showed that these differences were not statistically significant. However, significant differences were found between the pooled OKs (pooled mean 8.2 ± 2.3) and radicular cysts (p = 0.024). The variability of the number of layers in these two groups was also statistically significant so the radicular cyst images were more variable in the number of layers than the OKs images (Levene's test for Homogeneity of Variances, p = 0.032).

**Figure 3:**
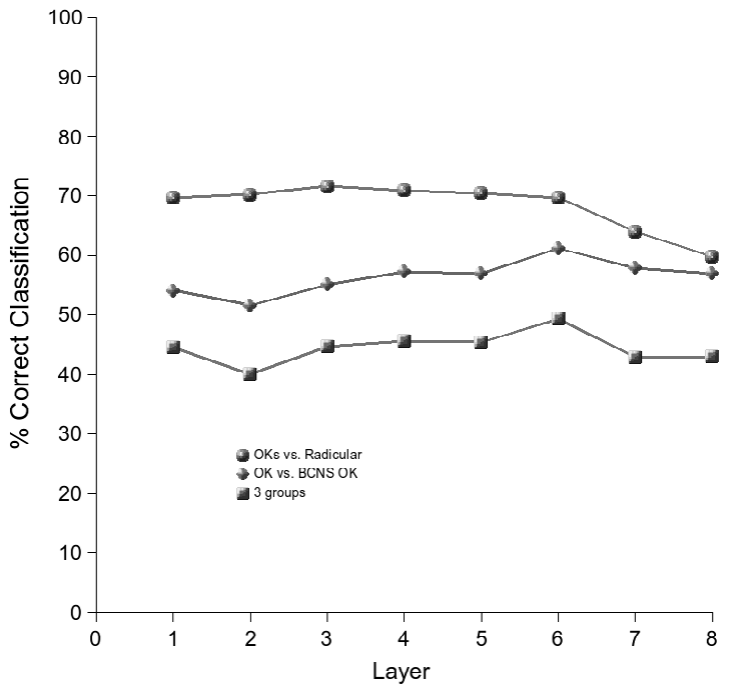
Layer-wise cell discrimination across the 3 types of cysts. The discrimination rates remain relatively consistent across layers. The largest discrimination is achieved between the (pooled) keratocysts and radicular cyst categories. **OK**: solitary odontogenic keratocysts, **Radicular**: radicular cysts, **BCNS OK**: Basal cell naevus syndrome keratocysts, **OKs**: keratocysts (pooled, solitary+syndrome), 3 groups: discrimination into any of the three groups (**OK **vs. **BCNS OK **vs.**Radicular**).

**Figure 4:**
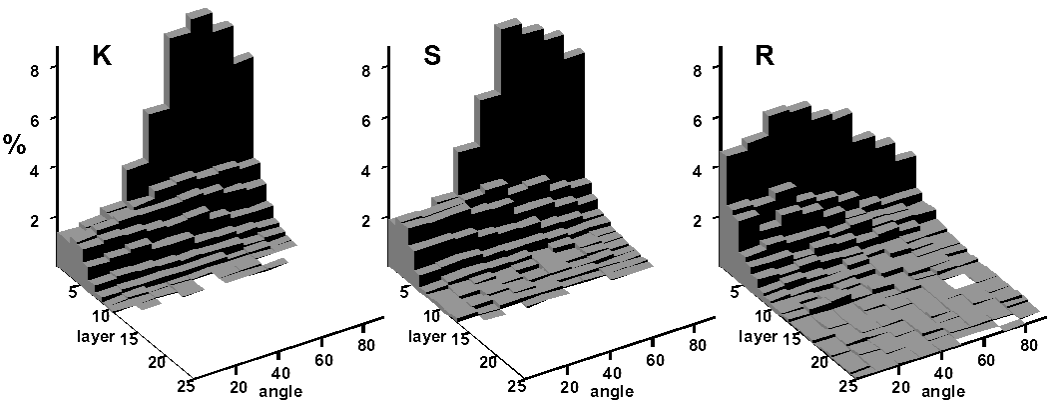
Distribution of angles of the major axis length of cells with respect to the layer orientation at the various layers of the cystic epithelial lining.**K**: solitary odontogenic keratocysts, **S**: basal cell naevus syndrome associated odontogenic keratocysts, **R**: radicular cysts. Note the differences in the distribution of layer 1 (the basal cell layer) across the keratocysts and radicular cysts and the tendency of radicular cysts to have more layers.

The layer-wise rates of correct classification of cells based on the morphological descriptors are shown in Figure 3. These rates are slightly improved, especially for the OKs vs. radicular cysts. The distribution of angles of the cell major axis length (Feret) per layer also provided an accurate illustration of the different architectures between the OKs and the radicular cysts. Figure 4 shows that these angles tend to approach an orthogonal direction in the first two layers and disappear in the upper layers. Traditionally this is known as cell palisading of the basal cell layer (layer 1 here) and it is characteristic of OKs, however this feature is absent in radicular cysts.

### Sample and case-wise comparisons

Sample-wise discrimination rates were also investigated based on the mean morphological values per sample. The correct discrimination into 3 classes across the 150 samples was 66% (cross-validated values were 59, 60 and 82% for the solitary OK, syndrome OK and radicular cysts, respectively). These figures showed that the differences between the 2 subtypes of OK, although statistically significant, were not sufficient for classification, however when the two OK subtypes were pooled together, the rate of correct classification was increased to 95%.

Case-wise, out of the 30 cases (with 5 images per case), only 1 case of radicular cyst had a majority of images wrongly classified as OK, corresponding to a 3.3% error rate.

## Discussion

Although the histological differences between radicular and OKs are usually enough to allow histopathologists to reach a definite diagnosis, the differences between OK subtypes remains an unresolved issue. For this reason, the purpose of this paper was directed to quantify the histomorphological differences in the epithelial lining architecture across the cyst types and to determine the power of discrimination (if any) that can be achieved using such quantitative markers.

The present study found that there were statistically significant differences in the epithelial architecture of OKs and radicular cysts and between the subtypes of OKs. Radicular cysts have on average more layers and their number varies more than in OKs. Furthermore, the discrimination rate achieved between OKs and radicular cysts samples (95%) was found to be higher than other previously published reports [3]. At the same time, rates for the discrimination between the two OKs subtypes, were not as high (around 60%), making them not suitable for detection of a BCNS case based on the cyst epithelial architecture alone. This poses an interesting question regarding the possibility of diagnosing BCNS cases in the light of other data published. For instance, Günhan et al [3] compared nuclear shape, nuclear size and DNA contents of the nuclei of OKs (without considering whether they were solitary or BCNS cysts) versus other odontogenic cysts (radicular and dentigerous) and reported statistically significant differences in the basal and intermediary cells. A more thorough analysis of the nuclear geometry of solitary and BCNS OKs was performed by Giardina et al. [2] who indicated that nuclear shape features (but not nuclear size) could be of diagnostic value (the discrimination rates, however, were not reported). Another study of 328 cysts (site-matched) found that a number of histological features (namely the number of satellite cysts, solid epithelial proliferations, ameloblastoma-like proliferations and odontogenic rests) were more commonly seen in syndrome cases [10]. Those features were indicative of increased cell proliferation rates which were later confirmed using counts of Ki-67 positive cells [5]. However, it seems that all the statistical differences reported are useful to differentiate between populations, but they do not guarantee a classifier for individual observations (obviously these are two different problems).

A possible explanation for the lack of definitive morphological markers for BCNS OKs may relate to their aetiology: it has been observed that genetic abnormalities (mutations and loss of heterozygosity) of common tumour suppressor genes, including the drosophila-homologous *Patched *gene (PTCH) are associated with the BCNS (as well as some other epithelial tumours, such as basal cell carcinomas). These abnormalities tend to be also present in both subtypes of OKs [1,8] and seem to be essential for the formation of such lesions. It is therefore possible that syndrome and solitary OKs are just two aspects of a single mechanism acting at different levels. The differences observed between OKs may be due to the degree and type of the genetic abnormality (several mutations were previously reported [1,8]) rather than being two distinct morphological entities. This may be eventually clarified by genetic analysis of non-cystic cells in patients with solitary OKs. One possibility is that while BCNS patients have widespread genetic abnormalities of the PTC gene throughout the tissues (therefore the multiple affections of the syndrome) the solitary patients may have similar abnormalities distributed on a much smaller scale (similarly to the cell distribution patterns found in unbalanced genetic mosaics and chimaeras [4]) or even limited to single clonal lines harbouring mutations which occurred late in development. Identifying which tissues are affected by the genetic abnormalities and to what degree, may provide further understanding of the disease development in non-syndrome patients.

Despite the large number of cells analysed in this work (27,806), a limited number of cases were studied. The analysis of more samples, including other types of cysts, and more importantly OKs with secondary inflammatory infiltration, may clarify to which extent the discrimination rates are retained (since it is a well established fact that secondarily inflamed OKs loose their characteristic lining and can resemble other inflammatory cysts).

Finally, appropriate characterisation of the lining in cystic lesions may also help to better understand their growth. It is only recently that the behaviour of epithelial cysts has been mathematically modelled [14]. Obviously these models are abstractions of natural processes which are based on quantitative characterisation of features which, in turn, are translated into numerical constants used by the model. Precise quantitative information such as presented here is likely to allow those models to become more accurate in terms of outcome prediction and validation.

## Conclusion

The measures of epithelial architecture presented can quantify in an unbiased manner the morphological characteristics of epithelial cyst linings. These measures provide an extra level of hierarchical description of the tissue make up that individual cell morphology alone cannot provide. Such analytical approach allows a high (case-wise 97% correct) discrimination between radicular and odontogenic keratocyst linings. However the differences between solitary and syndromic keratocysts do not allow discrimination of the syndrome based solely on the histological appearance of the tissues.

## List of abbreviations

ANOVA: analysis of variance

BCNS: basal cell naevus syndrome

H&E: haematoxylin and eosin

OK: odontogenic keratocyst

PTCH: patched (gene)

## Competing interests

The author(s) declare that they have no competing interests.
